# Peritonitis Due to Streptococcus uberis in a Patient on Automated Peritoneal Dialysis: A Case Report and Literature Review

**DOI:** 10.7759/cureus.97253

**Published:** 2025-11-19

**Authors:** Gennaro Argentino, Maria Luisa Sirico, Alessandra Antonia Mele, Lucia Di Micco, Mario Iorio, Andrea Pota, Vita Dora Iula, Andrea Camocardi

**Affiliations:** 1 Department of Nephrology and Dialysis, Ospedale del Mare, Naples, ITA; 2 Department of Clinical Pathology, Ospedale del Mare, Naples, ITA

**Keywords:** antimicrobial resistance, automated peritoneal dialysis, maldi-tof, peritoneal dialysis, peritonitis, soda gene, streptococcus uberis, zoonosis

## Abstract

Peritoneal dialysis (PD)-related peritonitis remains a major cause of morbidity, technique failure, and hospitalization in patients receiving renal replacement therapy. While *Staphylococcus* species and *Enterobacterales* represent the predominant etiologies, emerging reports have described rare infections caused by environmental or zoonotic organisms. *Streptococcus uberis*, a bovine mastitis pathogen and ubiquitous environmental microorganism, has been only exceptionally associated with human disease.

We report a case of peritonitis due to *S. uberis* in a 66-year-old male receiving automated peritoneal dialysis (APD; HomeChoice™ Pro Cycler, Baxter Healthcare, Deerfield, IL). The diagnosis was established using matrix-assisted laser desorption/ionization time-of-flight mass spectrometry (MALDI-TOF MS; Bruker Biotyper®, Bruker Daltonics, Bremen, Germany) and confirmed by sequencing of the *sodA* gene. Antimicrobial susceptibility testing (Etest®, bioMérieux, Marcy-l’Étoile, France) demonstrated susceptibility to β-lactams and glycopeptides.

The patient was successfully treated with a 21-day course of intraperitoneal vancomycin and ceftazidime, with complete clinical resolution and no relapse at 18-month follow-up. This case expands the spectrum of pathogens implicated in PD-associated infections and highlights the role of advanced microbiological diagnostics in identifying uncommon organisms. Environmental exposure should be considered in PD patients presenting with atypical or culture-negative peritonitis.

## Introduction

Peritonitis remains one of the most significant complications in patients undergoing peritoneal dialysis (PD), contributing to catheter loss, transition to hemodialysis, hospitalization, and mortality. Despite improvements in connection systems, patient training, and aseptic technique, PD-related infections persist as a major barrier to long-term technique survival. The microbiological spectrum is dominated by Gram-positive organisms, particularly *Staphylococcus epidermidis*, *Staphylococcus aureus*, and other coagulase-negative staphylococci, which account for approximately 60-70% of cases. Gram-negative organisms and fungi are less common but frequently associated with adverse outcomes.

Increasingly sensitive diagnostic technologies, including matrix-assisted laser desorption/ionization time-of-flight mass spectrometry (MALDI-TOF MS; Bruker Biotyper®, Bruker Daltonics, Bremen, Germany), broad-range 16S rRNA gene polymerase chain reaction (PCR), and housekeeping-gene sequencing (e.g., sodA and rpoB), have expanded the ability to detect unusual organisms that were previously misidentified by conventional microbiology.

*Streptococcus uberis* is an environmental and bovine mastitis pathogen, commonly isolated from soil, livestock bedding, and water. Human infections are extremely rare and typically limited to case reports, most involving ocular, urinary, or soft-tissue infections. Its identification can be challenging, as phenotypic methods may misclassify it as other viridans-group streptococci. Additionally, antimicrobial susceptibility patterns may differ from related species, and evidence-based interpretive breakpoints remain limited.

We present a case of peritonitis due to *S. uberis* in a patient treated with automated peritoneal dialysis (APD; HomeChoice™ Pro Cycler, Baxter Healthcare, Deerfield, IL). This case adds to the limited body of literature on human infections caused by this organism and emphasizes the critical role of advanced diagnostics and environmental exposure assessment in PD-related infections.

## Case presentation

A 66-year-old Caucasian male with end-stage renal disease secondary to hypertensive nephrosclerosis had been on continuous cycling peritoneal dialysis (CCPD) for 24 months using a HomeChoice™ Pro Cycler (Baxter Healthcare, Deerfield, IL, USA). His regimen consisted of four overnight exchanges with 2.0 L of 1.36% glucose-based dialysate and a daytime dwell, with no prior episodes of peritonitis.

His medical history included hypertension and hyperuricemia, treated with amlodipine, irbesartan, and allopurinol. He denied recent antibiotic use, abdominal procedures, or catheter malfunction. He reported a recent rural trip to Campania, Italy, where he had indirect exposure to livestock barns housing cattle and water buffalo.

He presented with cloudy peritoneal effluent, mild abdominal pain, and low-grade fever (38.5°C). Examination revealed mild diffuse abdominal tenderness without guarding, and a clean exit site without erythema or drainage. Laboratory results are shown in Table 1. Peritoneal fluid contained 4,370 white blood cells (WBC)/mm³ (80% polymorphonuclear neutrophils (PMN)), fulfilling the International Society for Peritoneal Dialysis (ISPD) 2022 criteria for peritonitis.

**Table 1:** Baseline laboratory parameters.

Parameter	Value	Reference range
White blood cells	13,410/mm³ (90.7% neutrophils)	4,000–10,000/mm³
C-reactive protein	6.3 mg/dL	<0.5 mg/dL
Procalcitonin	0.77 ng/mL	<0.05 ng/mL
Serum creatinine	5.4 mg/dL	0.6–1.3 mg/dL
Blood urea nitrogen	72 mg/dL	7–20 mg/dL
Total protein	6.7 g/dL	6.0–8.0 g/dL
Fasting glucose	94 mg/dL	70–110 mg/dL
Total cholesterol	182 mg/dL	<200 mg/dL
High-density lipoprotein cholesterol	48 mg/dL	>40 mg/dL
Low-density lipoprotein cholesterol	112 mg/dL	<130 mg/dL
Triglycerides	124 mg/dL	<150 mg/dL

Empirical intraperitoneal vancomycin (30 mg/kg, full-dwell intermittent dosing) and ceftazidime (250 mg/L) were started. Dialysate samples were inoculated into aerobic and anaerobic blood culture bottles (BACTEC™, Becton Dickinson, Franklin Lakes, NJ). Within 48 hours, aerobic cultures were positive. Colonies were catalase-negative and α-hemolytic. MALDI-TOF MS (Bruker Biotyper®, Bruker Daltonics) identified *Streptococcus uberis* with a high-confidence score. Species confirmation was achieved by *sodA* gene sequencing (430 bp amplicon, 100% identity to reference genome). Antimicrobial susceptibility testing was performed using Etest® (bioMérieux, Marcy-l’Étoile, France). The clinical course and therapeutic steps are shown in Table 2, while the detailed antimicrobial susceptibility profile of the isolate is reported in Table 3. The patient became afebrile within 72 hours, and dialysate clarity normalized by day five. All subsequent peritoneal cultures remained sterile. Therapy was continued for 21 days. The patient remained free of recurrence at 18-month follow-up.

**Table 2:** Therapeutic timeline and clinical course. Therapeutic timeline summarizing the patient’s clinical course, microbiological milestones, and response to intraperitoneal therapy. AST: antimicrobial susceptibility testing; IE: insufficient evidence (EUCAST v.13.0); IP: intraperitoneal; PD: peritoneal dialysis; PMN: polymorphonuclear neutrophils; WBC: white blood cells; MALDI-TOF MS: matrix-assisted laser desorption/ionization time-of-flight mass spectrometry.

Day	Clinical/laboratory findings	Intervention/remarks
0	Onset of cloudy effluent, mild abdominal pain, and fever of 38.5°C. Effluent WBC 4,370/mm³ (80% PMN).	Empirical IP antibiotics started: vancomycin 30 mg/kg (all bags; trough 15–20 µg/mL) + ceftazidime 250 mg/L of dialysate.
2	Cultures were positive (aerobic bottles). Catalase-negative α-hemolytic colonies.	MALDI-TOF MS identification: *Streptococcus uberis* (log > 2.2).
3	sodA gene PCR/sequencing confirms 100% identity (CP009999).	AST (Etest, EUCAST v.13.0): susceptible to β-lactams, vancomycin; linezolid: resistant; macrolides/aminoglycosides: IE.
3–5	Rapid clinical improvement: afebrile by 72 hours, pain resolution, effluent clearing.	Continuation of the same regimen; no adverse effects.
5–14	Effluent WBC < 100/mm³; culture-negative.	Therapy continued to complete a full 21-day course (both agents) to ensure Gram-negative coverage and prevent relapse.
7–21	Repeated effluent cultures were sterile.	Full recovery; no catheter malfunction or exit-site infection.
>18 months follow-up	No recurrence; stable PD technique and peritoneal function.	-

**Table 3:** Antimicrobial susceptibility profile of Streptococcus uberis isolate (Etest, EUCAST v.13.0). AST by Etest (bioMérieux®). Interpretation per EUCAST v.13.0 for viridans-group streptococci. QC strain: *Streptococcus pneumoniae* ATCC 49619. IE: insufficient evidence to establish species-specific clinical breakpoints. S/I/R categories apply where EUCAST provides breakpoints for related viridans-group streptococci. MIC: minimum inhibitory concentration.

Antibiotic	MIC (mg/L)	Category (EUCAST v.13.0)
β-lactams		
Ampicillin	≤0.25	S
Benzylpenicillin	0.25	S
Cefotaxime	≤0.12	S
Ceftriaxone	0.5	S
Macrolide/lincosamide		
Erythromycin	>4	IE
Clindamycin	>0.5	R
Fluoroquinolones		
Levofloxacin	0.5	IE
Moxifloxacin	0.12	IE
Oxazolidinone		
Linezolid	2	IE
Glycopeptides		
Teicoplanin	≤0.12	S
Vancomycin	0.5	S

## Discussion

PD-related peritonitis has been extensively described in the literature, with Gram-positive organisms representing the majority of episodes [1-6], a significant contribution of Gram-negative bacteria to severe clinical outcomes [3], and a broader microbiological spectrum that includes emerging environmental or veterinary-associated pathogens [7-9]. This case highlights an uncommon etiology of PD-related peritonitis caused by *Streptococcus uberis*, traditionally regarded as a bovine mastitis pathogen and environmental microorganism.

Microbiological and taxonomic considerations

*Streptococcus uberis* belongs to the viridans-like streptococci group, a heterogeneous cluster frequently misidentified by conventional microbiology [6,10]. In this case, initial cultures showed catalase-negative, α-hemolytic colonies. MALDI-TOF MS (Bruker Biotyper®, Bruker Daltonics) enabled high-confidence identification of *S. uberis*, later confirmed by sequencing of the *sodA* gene [6]. The complementary use of proteomic and molecular methods reflects best practice for accurate species-level identification of viridans-group streptococci [5,6].

Clinical course and therapeutic implications

The clinical presentation, i.e., cloudy effluent, abdominal discomfort, and elevated peritoneal white blood cell (WBC) count, was typical of PD-related peritonitis. Empirical intraperitoneal vancomycin and ceftazidime were started according to the ISPD guidelines [1]. Antimicrobial susceptibility testing demonstrated susceptibility to β-lactams and glycopeptides, consistent with patterns described in veterinary isolates [9] and in rare reported human infections. The patient experienced rapid improvement and remained recurrence-free at 18-month follow-up. The therapeutic timeline is shown in Table 2, and antimicrobial susceptibility in Table 3.

Previously reported human infections due to *Streptococcus uberis*

Human infections caused by *S. uberis* remain rare and have included intraocular endophthalmitis, urinary tract infections [11,12], community-acquired pneumonia [13], and a soft-tissue infection with bloodstream involvement in a hemodialysis patient [14]. These reports are summarized in Table 4 and classified by infection type and diagnostic method in Table 5.

**Table 4:** Summary of published human cases of S. uberis infection. This table includes only documented human cases with published data and confirmed bacterial identification. Animal studies and unverified or suspected reports were excluded. MALDI-TOF MS: matrix-assisted laser desorption/ionization time-of-flight mass spectrometry.

Year	Author (reference)	Clinical presentation	Host factors/comorbidities	Isolation site	Identification method	Outcome
2010	Velez-Montoya et al. [12]	Endophthalmitis (intra-ocular)	Postoperative ocular surgery	Vitreous humor	Culture + unspecified antibiotic-resistance study	Recovery after intravitreal ampicillin + other therapy
2013	Gülen et al. [11]	Urinary tract infections	Various patients (agricultural/rural exposure)	Urine	Biochemical phenotypic identification	Resolution after therapy in most cases
2015	Valentiny et al. [14]	Soft tissue infection + bacteremia	Hemodialysis; diabetic foot	Wound, blood	MALDI-TOF MS (molecular)	Recovery after combined therapy

**Table 5:** Classification of reported Streptococcus uberis human infections by type, year, and clinical context. This table was derived strictly from the currently cited references in the manuscript [11-14] plus the present case. Categories unsupported by these references were not included. Animal studies and unverified reports were excluded. CAP: community-acquired pneumonia; ESRD: end-stage renal disease; PD: peritoneal dialysis; MALDI-TOF MS: matrix-assisted laser desorption/ionization time-of-flight mass spectrometry.

Infection type	Number of cases	Year range	Main clinical contexts	Predisposing factors	Typical diagnostic approach
Ocular – endophthalmitis [12]	1	2010	Postoperative intra-ocular infection	Ocular surgery (post-op)	Culture (vitreous); targeted intravitreal therapy reported
Soft tissue + bacteremia [14]	1	2015	Diabetic foot; hemodialysis patient	Diabetes; hemodialysis	MALDI-TOF MS (species ID); wound/blood culture
Urinary tract infection [11]	7	2013	Community-acquired UTIs	Various; several with probable environmental exposure	Urine culture; phenotypic/biochemical identification
Community-acquired pneumonia (conference abstract) [13]	1	2012	CAP (meeting abstract)	Not reported	Reported in abstract; limited diagnostic detail
Peritonitis – current case	1	2025	Peritoneal dialysis	ESRD; PD catheter exposure	Culture + MALDI-TOF + *sodA* gene sequencing (as performed)

**Figure 1:**
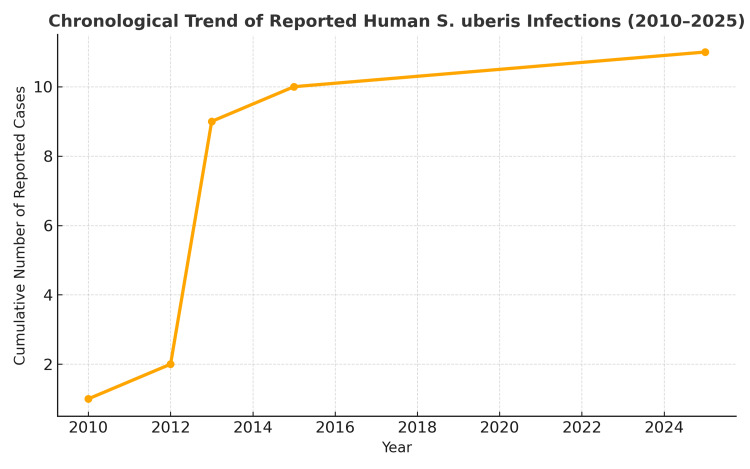
Chronological trend of reported human Streptococcus uberis infections (2010–2025). Cumulative number of microbiologically confirmed human infections due to *Streptococcus uberis* reported in the literature from 2010 to 2025, derived from published cases [11-14] and the present report. Each point represents the cumulative number of documented cases up to that year: intraocular infection (endophthalmitis, 2010) [12], community-acquired pneumonia (2012) [13], seven urinary tract infections (2013) [11], a soft-tissue and bloodstream infection in a hemodialysis patient (2015) [14], and peritoneal dialysis–related peritonitis (2025, current case).

A chronological overview of confirmed human infections, including the present case, is illustrated in Figure 1, highlighting the progressive expansion of clinical presentations and the increasing adoption of advanced microbial diagnostics [11-14].

Environmental exposure and One Health considerations

*S. uberis* is a major cause of environmental mastitis in cattle, commonly associated with soil, bedding, and animal housing [9]. The patient’s recent indirect exposure to livestock barns in southern Italy suggests possible environmental acquisition, consistent with previous reports involving agricultural or environmental contact [11-13]. This aligns with the One Health framework, emphasizing the interconnectedness of human, animal, and environmental health domains [15]. PD patients with rural or agricultural exposure may benefit from targeted hygiene and catheter care counseling to reduce the risk of environmental peritonitis [14,15].

Diagnostic and laboratory considerations

Key priorities for PD programs and microbiology laboratories include the following: routine use of MALDI-TOF MS to enhance detection of uncommon pathogens [5,10]; molecular confirmation (e.g., sodA and rpoB) when MALDI-TOF identifies atypical species [6]; standardized antimicrobial susceptibility reporting, including EUCAST version, method, and quality control (QC) strain, to improve comparability [9]; comprehensive reporting of diagnostic methods to support recognition of rare pathogens.

Alignment with the ISPD recommendations and clinical practice

Management in this case followed the ISPD guidance, emphasizing rapid empirical treatment, culture-directed adjustments, and appropriate treatment duration [1]. The absence of recurrence over long-term follow-up supports the adequacy of this approach. This case underscores the need for broad differential diagnosis and early use of advanced microbiological tools in PD-related peritonitis, particularly in patients with environmental exposure.

## Conclusions

PD-related peritonitis is most commonly caused by skin or enteric flora, but this case demonstrates that environmental streptococci such as *Streptococcus uberis* can also act as opportunistic pathogens in selected patients. Advanced microbiological techniques, including MALDI-TOF MS and housekeeping-gene sequencing, were crucial for accurate species-level identification in this setting and allowed effective, targeted intraperitoneal therapy with vancomycin and ceftazidime, resulting in sustained clinical cure. Clinicians should remain alert to atypical organisms in PD effluent, particularly in the presence of environmental exposures or unexpected susceptibility patterns.

This case also highlights the relevance of the One Health concept in the care of PD patients, emphasizing the interplay between human, animal, and environmental health in the emergence of rare infections. Detailed environmental histories, appropriate counseling regarding rural and agricultural exposures, and close collaboration between nephrologists, microbiologists, and public health professionals can enhance prevention, early recognition, and optimal management of uncommon PD-related infections.
